# The performance of interferon-gamma release assay in nontuberculous mycobacterial diseases: a retrospective study in China

**DOI:** 10.1186/s12890-016-0320-3

**Published:** 2016-11-25

**Authors:** Mao-Shui Wang, Jun-Li Wang, Xin-Feng Wang

**Affiliations:** 1 Department of Lab Medicine, Shandong Provincial Chest Hospital, 46# Lishan Road, Jinan City, 250013 People’s Republic of China; 2 Center of Clinical Laboratory, Affiliated Hospital of Youjiang Medical College for Nationalities, Baise, China

**Keywords:** T-SPOT.TB, Nontuberculous mycobacterium infections, Tuberculosis, Mixed infection

## Abstract

**Background:**

The interferon-gamma release assay (IGRA) is more specific than the tuberculin skin test to discriminate between tuberculosis (TB) and nontuberculous mycobacterial (NTM) diseases. Here we performed a retrospective study to evaluate the performance of the T-SPOT.TB in patients with NTM diseases.

**Methods:**

Between March, 2013 and Nov, 2015, a total of 58 patients with NTM diseases had a T-SPOT.TB performed were enrolled, 30 patients had definite NTM diseases, 28 had probable diseases. Their clinicopathological characteristics were reviewed and analyzed. Cultures for mycobacteria were performed. The indirect proportion method with Löwenstein–Jensen (L-J) medium was used for first-line drug susceptibility test. T-SPOT.TB assay was performed according to the manufacturer’s instructions. Data were expressed as mean ± standard deviation (continuous variables) and as numbers and percentages (categorical variables). The *χ* ^2^ test was used for comparisons between proportions.

**Results:**

The average age was 51.8 ± 16.1 years (range 10 to 77 years), 58.6% (34/58) were male. 16.4% (9/55) were TB-PCR positive. 34 (58.6%) isolates were *Mycobacterium intracellulare*, ten (17.2%) were *Mycobacterium chelonae* and seven (12.1%) were *Mycobacterium fortuitum*. Fifty-two (89.7%) patients were NTM lung disease, five (8.6%) were pleural disease, and one (1.7%) lymphadenitis. The total positivity of T-SPOT.TB was 53.4% (31/58) among the whole group (probable and definite). For probable cases, the T-SPOT.TB assay was positive in 53.5% (15/28); for definite cases, 16 (53.3%) of 30 definite cases were positive. There was no statistical difference in the positivity rate between them (*P* < 0.01).

**Conclusions:**

In the study, we showed that a significant portion of NTM diseases were T-SPOT.TB positive in China. Although T-SPOT.TB is useful diagnostic method for differentiating TB from NTM diseases, in China, the IGRA assay show limited value in the discrimination. In addition, further research is needed to investigate the association between TB infection and treatment for NTM patients.

## Background

The nontuberculous mycobacteria (NTM) are ubiquitous microorganisms found in various environments [1]. NTM can cause both asymptomatic infection and symptomatic disease, lung is the most common site of NTM infection. It is noteworthy that the prevalence of NTM disease is increasingly reported worldwide [2]. In China, the most frequent causative organisms of NTM are *Mycobacterium intracellulare (81.2%)*, *Mycobacterium kansasii* (7.8%) and *Mycobacterium fortuitum* (4.7%) [3].

Currently, the lack of randomized clinical trials to guide treatment results in that treatment strategies are largely based on expert opinion [4, 5]. Meanwhile, the diagnosis of pulmonary NTM disease is significantly delayed in China [6]. NTM diseases share clinical signs with tuberculosis (TB), causing a clinical dilemma in differentiation between NTM infection and TB. Until now, the Tuberculin skin test (TST) remains to be used for detecting latent TB infection and an adjunctive test for active TB. However, the TST suffers from false positivity in NTM diseases and previous BCG-vaccination [7].

Interferon-gamma (IFN-γ) release assays (IGRAs), such as T-SPOT.TB, QuantiFERON-TB Gold Test (QFT-GT), are more specific and are based on the T cell mediated IFN-γ release after stimulation with specific *Mycobacterium tuberculosis* (*M.TB*) antigens. These tests have a better specificity worldwide without cross-reactivity with most NTM [8] and BCG, and a higher sensitivity compared to the TST for detection of active TB or latent TB infection [9, 10]. The T-SPOT.TB assay is based on response to the *M.TB* specific peptide antigens ESAT-6 and CFP-10 which are located in the region of difference (RD1). The RD1 is present in mycobacteria belonging to the *M.TB* complex (*M.TB, Mycobacterium africanum, Mycobacterium bovis, Mycobacterium canettii, Mycobacterium caprae, Mycobacterium microti, Mycobacterium pinnipedii, Mycobacterium mungi and Mycobacterium orygis*) [11] and very few NTM species also share the RD1 of *M.TB* complex (*Mycobacterium gastri, Mycobacterium kansasii, Mycobacterium marinum, Mycobacterium riyadhense* and *Mycobacterium szulgai*) [8, 12, 13]. Therefore, infection with these strains may result in a positive result of T-SPOT.TB assay. Due to the high specificity of the IGRAs, some studies have suggested that it can be used to discriminate between infection with TB and NTM [14–17].

The goal of this retrospective study is to evaluate the performance of the T-SPOT.TB in patients with NTM diseases in a high TB-burden country.

## Methods

The protocol was approved by the Ethical Committee of Shandong Provincial Chest Hospital, written informed consent was not required because of the retrospective nature of the investigation.

Between March, 2013 and Nov, 2015, a total of 58 patients with NTM diseases had a T-SPOT.TB performed were enrolled, 30 patients had definite NTM diseases, 28 had probable diseases. Their clinicopathological characteristics were reviewed and analyzed.

Cases were defined as “definite” according to the 2007 ATS/IDSA criteria for disease when they had clinical criteria and there were at least two separate positive cultures of sputum samples, or at least one positive culture from bronchial lavage or lung biopsy [1]. Patients with a positive NTM culture with a compatible clinical syndrome were termed as “probable”. Cultures for mycobacteria were performed using Löwenstein–Jensen medium (L-J) method. The indirect proportion method with L-J medium was used for first-line drug susceptibility test (DST) [18].

T-SPOT.TB assay (Oxford Immunote Ltd., Edinburgh, UK) was performed according to the manufacturer’s instructions. Briefly, peripheral blood mononuclear cells was separated from a whole blood sample and incubated with the antigens (ESAT-6 and CFP10). The secreted cytokine by sensitized T cell was captured by specific antibodies on the membrane. Finally, the cytokine was detected by a chromogenic spot assay. Following manufacture instructions, the result of the testing was categorized “positive” or “negative” by spot count.

Statistical analysis was carried out using SPSS 17.0 software. Data were expressed as mean ± standard deviation (continuous variables) and as numbers and percentages (categorical variables). The *χ* ^2^ test was used for comparisons between proportions. All statistical tests are 2-sided at α = 0.05.

## Results

Table 1 shows the characteristics of the participants of this study. The average age was 51.8 ± 16.1 years (range 10 to 77 years), 58.6% (34/58) were male. One hundred percent (51/51) were HIV-negative. 16.4% (9/55) were TB-PCR positive. 50.9% (29/57) were positive for acid-fast bacillus staining. NTM were cultured from sputum (84.5%), pleural effusion (8.6%), bronchial brushing (5.2%) and tissues (1.7%). Thirty-four (58.6%) isolates were *Mycobacterium intracellulare*, ten (17.2%) were *Mycobacterium chelonae*, seven (12.1%) were *Mycobacterium fortuitum*, four (6.9%) were *Mycobacterium kansasii*, one (1.7%) were *Mycobacterium avium*, one (1.7%) were *Mycobacterium scrofulaceum* and one (1.7%) were *Mycobacterium terrae*. Phenotypic DST was performed on 49 isolates, 48 (98.0%) isolates were resistant to isoniazid, 44 (89.8%) resistant to rifampin, 43 (87.8%) resistant to streptomycin, 26 (53.1%) resistant to ethambutol and 24 (49.0%) resistant to the four drugs.

**Table 1:** Demographic and clinical characteristics of probale and definite NTM patients

	Probable (*n* = 28)	Definite (*n* = 30)	Total (*n* = 58)
Age (years)	55.8 ± 13.1	48.0 ± 17.9	51.8 ± 16.1
Sex, male	53.5% (15)	63.3% (19)	58.6% (34/58)
HIV (+)	100% (25/25)	100% (26/26)	100% (51/51)
TB-PCR (+)	10.7% (3/28)	22.2% (6/27)	16.4% (9/55)
AFB staining (+)	63.0% (17/27)	40% (12/30)	50.9% (29/57)
NTM strains			
*Mycobacterium intracellulare*	60.7% (17)	56.7% (17)	58.6% (34/58)
*Mycobacterium chelonae*	17.9% (5)	16.7% (5)	17.2% (10/58)
*Mycobacterium fortuitum*	10.7% (3)	13.3% (4)	12.1% (7/58)
*Mycobacterium kansasii*	10.7% (3)	3.3% (1)	6.9% (4/58)
*Mycobacterium avium*	0	3.3% (1)	1.7% (1/58)
*Mycobacterium terrae*	0	3.3% (1)	1.7% (1/58)
*Mycobacterium scrofulaceum*	0	3.3% (1)	1.7% (1/58)
DST (resistance)			
Isoniazid (1 μg/ml)	95.2% (20/21)	100% (28/28)	98.0% (48/49)
Rifampin (50 μg/ml)	85.7% (18/21)	92.9% (26/28)	89.8% (44/49)
Streptomycin (10 μg/ml)	90.5% (19/21)	85.7% (24/28)	87.8% (43/49)
Ethambutol (5 μg/ml)	47.6% (10/21)	57.1% (16/28)	53.1% (26/49)
Firsty-line drugs	42.9% (9/21)	53.5% (15/28)	49.0% (24/49)
Symptoms			
Cough	67.9% (19)	60.0% (18)	63.8% (37)
Fever	53.6% (15)	30.0% (9)	41.4% (24)
Dyspnea	25.0% (7)	36.7% (11)	31.0% (18)
Hemoptysis	7.1% (2)	10.0% (3)	8.6% (5)
T-SPOT.TB (+)	53.5% (15/28)	53.3% (16/30)	53.4% (31/58)

*HIV* human immunodeficiency virus, *TB-PCR* tuberculosis-polymerase chain reaction, *AFB* acid fast bacilli, *NTM* nontuberculous mycobacteria, *DST* drug susceptibility test

Fifty-two (89.7%) patients were NTM lung disease, five (8.6%) were pleural disease, and one (1.7%) lymphadenitis. Thirty-seven (63.8%) patients had cough, 24 (41.4%) fever, 18 (31.0%) dyspnea and five (8.6%) hemoptysis. Eight (13.8%) patients were asymptomatic and admitted for abnormality of chest X-ray. Seven patients have contact history with a TB patient in the family.

The total positivity of T-SPOT.TB was 53.4% (31/58) among the NTM group (probable and definite). For probable cases, the T-SPOT.TB assay was positive in 53.5% (15/28); for definite cases, 16 (53.3%) of 30 definite cases were positive; there was no statistical difference in the positivity rate between them (*P* < 0.01). For NTM strains, 47.1% (16/34) of *Mycobacterium intracellulare* cases were positive on T-SPOT.TB assay (seven were probable, nine definite); 70% (7/10) of *Mycobacterium chelonae* cases were T-SPOT.TB positive (three were probable, four definite); 57.1% (4/7) of *Mycobacterium fortuitum* cases were T-SPOT.TB positive (all were definite).

## Discussion

China, as one of the 22 high TB-burden countries, *M.TB* remains to be the main cause of pulmonary mycobacteria diseases. Differentiation between NTM infection and TB is very difficult. Since IGRAs has the potential to different TB from NTM diseases, we aimed to evaluate the use of T-SPOT.TB for the discrimination in China. Surprisingly, in the study, a high proportion of NTM patients were found to be T-SPOT.TB positive. The result implied that, 1) TB infection among NTM diseases was common in China; 2) T-SPOT.TB have limited value in discrimination of NTM diseases and TB in China, so the diagnosis of NTM diseases remains to rely on routine methods (such as culture). To best of our knowledge, this is the first report for evaluating the performance of IGRAs in NTM disease in the high TB-burden country.

It was previously thought that each patient get one kind of diseases (NTM or TB). Nonetheless, the development of immunological and molecular methods has led to increasing reports of mixed infection. Recently, several studies have reported the occurrence of mixed infection of TB and NTM [19–22]. In the study, we also found that 53.4% patients were T-SPOT.TB positive, and 16.4% NTM patients were TB-PCR positive. However, a study conducted at our hospital showed that, in the health care workers, the overall positivity rate of T-SPOT.TB test was 13.9% (122 out of 879; 95% CI 11.7–16.3%) [23]. Moreover, in a positive control group, including 238 culture confirmed TB patients (aged 39.1 ± 19.5, 64.7% were male), the positivity of T-SPOT.TB was 86.1% (205/238), our further statistical analysis showed the positivity of T-SPOT.TB in NTM diseases is lower than in positive control (data not published). Therefore, our findings should be a direct proof for the mixed infection. The coexistence of TB and NTM has very important clinical significance in some patients, mixed infection may be one of the reasons that may lead to failure in therapy of NTM diseases, so caution should be exercised [21]. For example, there were five patients (8.6%) were retreatment NTM disease in our study. Usually, NTM isolates were resistant to the first-line anti-TB drug. Therefore, these drugs wouldn’t be the first choice for treatment of TB patients. But now, since the co-infection exists, these drugs sensitive for TB but resistant for NTM stains should be reconsidered. Moreover, an unaccurate diagnosis of NTM diseases can be made due to the presence of NTM, which sometimes leads to inappropriate treatment [21, 24]. In addition, mixed TB and NTM may also present clinical problems for culture and DST, such as failure to recognize TB, over-estimation severity of drug resistance.

Kendall et al. reported that a significant portion of pulmonary TB patients were identified to have NTM, particularly in patients with cavitation and those born in the US [25]. Another US study identified 11.0% of pulmonary TB patients with NTM species [26]. Simultaneous detection of *M.TB* and NTM in a specimen is possible because NTM may be present as colonizers or as pathogens and because NTM is ubiquitous in the environment and is isolated frequently in immunocompromised patients, such as HIV-infected individuals and patients on immunosuppressive treatment (for example, Anti-TNF drugs, corticosteroids) [27]. Thus, an analysis was performed by Hwang SM et al., it was reported that a significant portion of NTM patients were identified to have TB, ten patients (14.5%) had a history of TB, mixed cultures (NTM and *M.TB*) were present repeatedly in 12 patients (17.4%) [21]. This research should be the first report addressing the issues on mixed infection of TB and NTM.

For evaluating diagnostic role of IGRAs in discrimination of TB and NTM diseases, several studies have been performed in low TB- burden countries, it showed that the IGRAs hold potential to discriminate between NTM and TB infections. A Japanese study showed that QFT-GT had a mean sensitivity of 86% and a mean specificity of 94%, the test is a useful diagnostic method for differentiating between them [28]. Hermansen TS et al. found that QFT-GT was positive in 8% of patients with definite NTM disease, and in 4% of patients with definite disease infected with NTM without the RD1 region. [17]. But in our study, we found that a significant portion of NTM diseases were T-SPOT.TB positive in China. Similar result was reported in [14], but the author didn’t address the issue and contribute it to the cross-reactivity. It means that in China, the IGRA assay show limited value in the discrimination between TB and NTM diseases. In our study, six kinds of NTMs (*Mycobacterium intracellulare, Mycobacterium chelonae, Mycobacterium fortuitum, Mycobacterium kansasii, Mycobacterium avium* and *Mycobacterium terrae*) were isolated, only *Mycobacterium kansasii* was known to express ESAT-6 and CFP-10 [29].

Our study has some limitations that could have an impact on the results. Bias is more common in retrospective study. However, bias in this study was minimized by consecutive cases were enrolled, and patients have equal chance to participate in the study. The sample size was small, caution needed when interpreting the results. Finally current diagnostic methods couldn’t identify true active TB from these T-SPOT.TB or TB-PCR positive cases. Therefore, we have no further suggestion on how to choose appropriate regime for NTM cases with T-SPOT.TB or TB-PCR positive, but we think the first-line anti-TB drugs should be considered.

## Conclusions

In the study, we showed that a significant portion of NTM diseases were T-SPOT.TB positive in China. Although T-SPOT.TB was useful diagnostic method for differentiating TB from NTM diseases in low-TB burden countries, in China, the IGRA assay show limited value in the discrimination. In addition, further research is needed to investigate the association between TB infection and treatment for NTM diseases.

## Abbreviations

DST: Drug susceptibility test
IGRA: Interferon-gamma release assay
NTM: Nontuberculous mycobacteria
QFT-GT: QuantiFERON-TB gold test
RD: Region of difference
TB: Tuberculosis
TB-PCR: Tuberculosis-polymerase chain reaction
TNF: Tumor necrosis factor
TST: Tuberculin skin test
